# An Unusual Cause of Hypoxia: Ventricular Septal Defect, Pulmonary Artery Atresia, and Major Aortopulmonary Collaterals Diagnosed in the Adult Cardiac Catheterization Lab

**DOI:** 10.1155/2020/4726529

**Published:** 2020-01-25

**Authors:** Katia Bravo-Jaimes, Brian Walton, Poyee Tung, Richard W. Smalling

**Affiliations:** Division of Cardiology, University of Texas Health Science Center at Houston, Houston, Texas, USA

## Abstract

The association of pulmonary atresia, ventricular septal defect (VSD) and major aortopulmonary collaterals (MAPCA) is an extreme form of tetralogy of Fallot (TOF). It carries a high mortality risk if not intervened on during infancy with only 20% of unoperated patients surviving into adulthood. We present the case of a 40-year-old man who presented for evaluation prior to retinal surgery and was found to have hypoxia and a loud murmur. Cardiac catheterization was performed in the general catheterization laboratory, demonstrating a membranous VSD, pulmonary atresia, and MAPCA. We highlight the challenges and limitations that an adult interventional cardiologist may have when encountering these patients.

## 1. Case Report

**Figure 1:**
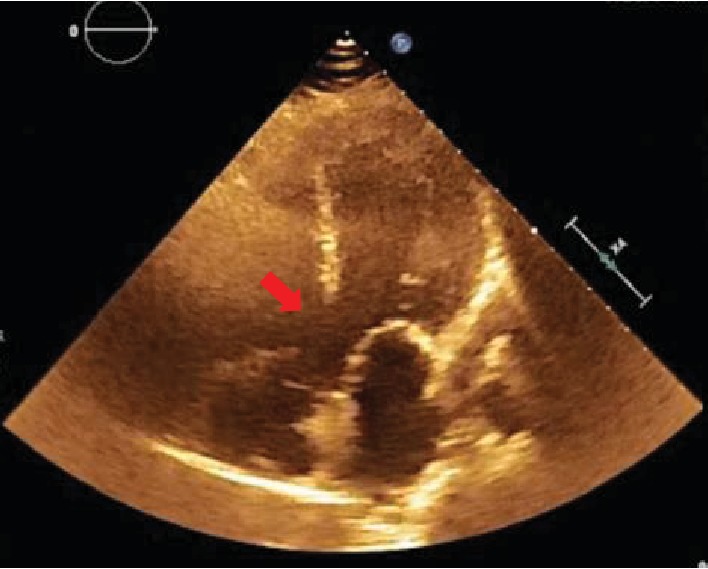
A five-chamber view showing an overriding aorta and ventricular septal defect (red arrow).

A 40-year-old man with scoliosis and developmental delay presented for outpatient evaluation prior to retinal surgery. His physical exam showed a heart rate of 110 bpm, blood pressure of 160/70 mmHg, respiratory rate of 16/minute, oxygen saturation of 84%, clubbing, elevated jugular venous pressure, and a multifocal V/VI systolic murmur radiating to the back. Laboratory tests were remarkable for hemoglobin of 18 g/dL. An echocardiogram demonstrated biventricular hypertrophy, preserved biventricular systolic function, an overriding aorta (Figure 1), and a right-to-left interventricular shunt.

**Figure 2:**
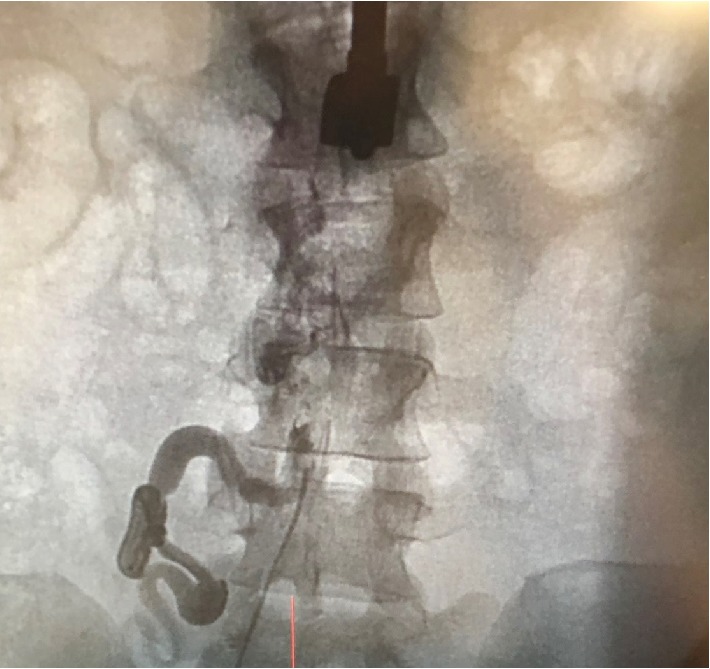
Interrupted inferior vena cava that prompted change from right femoral to right internal jugular access.

**Figure 3:**
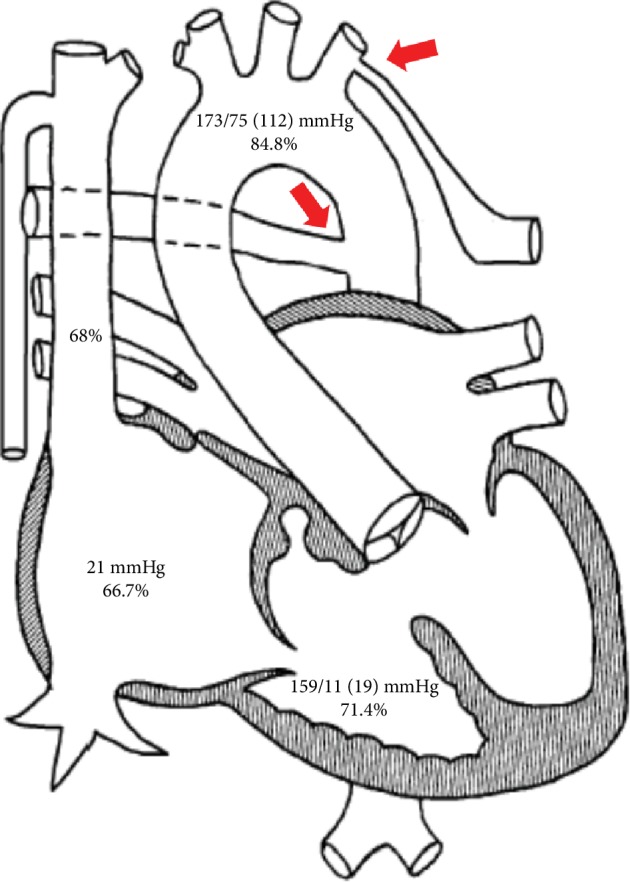
Diagram showing invasive hemodynamics and oximetry. Note that the right MAPCA originates from the descending aorta and the left MAPCA from the left subclavian artery (red arrows).

**Figure 4:**
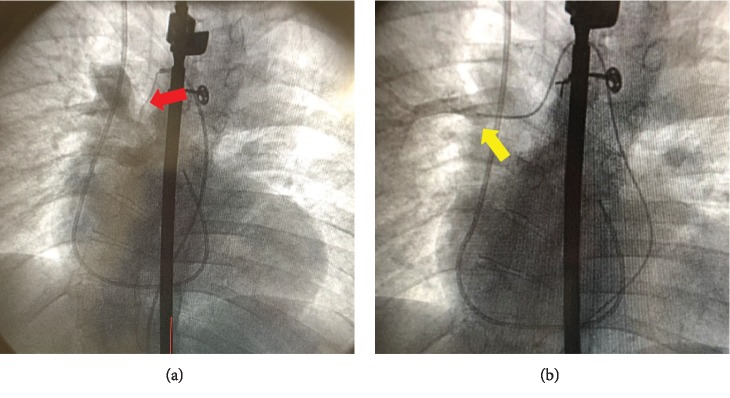
(a) Collaterals (red arrow) from the thoracic aorta. (b) After cannulation of this MAPCA, the right upper pulmonary artery branch was seen (yellow arrow) and had normal systolic pressure.

**Figure 5:**
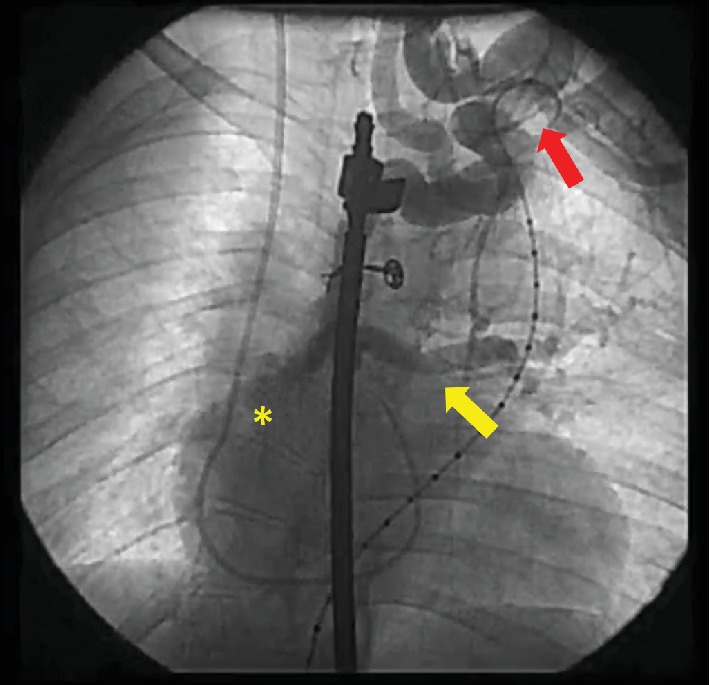
Collaterals (red arrow) from the left subclavian artery originating the left (yellow arrow) and right lower (asterisk) pulmonary artery branches.

A ventriculogram, right heart catheterization, and oximetry were performed. After initial femoral venous access, there was difficulty passing a J wire and angiography showed an interrupted inferior vena cava (Figure 2). Therefore, access was changed to the internal jugular vein and right heart catheterization demonstrated severely elevated right atrial and ventricular pressures (Figure 3). There was an inability to obtain pulmonary artery waveforms, and a right ventriculogram demonstrated pulmonary atresia. A pigtail catheter was introduced via femoral arterial access, and simultaneous right and left ventricular pressures were obtained. Ventriculography confirmed a membranous VSD. Subsequent root and thoracic aortography showed MAPCA from the lower thoracic aorta connecting to the right upper pulmonary artery branch (Figure 4, [Supplementary-material supplementary-material-1]). The main communication to the right lung was cannulated via the MAPCA, and a pulmonary angiogram was performed. There were two areas of stenosis. The pressures in the right upper pulmonary artery branch were measured at 120 mmHg proximal and 18 mmHg distal to the areas of stenosis. Next, the left subclavian artery was engaged, and an angiogram was performed, revealing MAPCA between the left subclavian artery and left and right lower pulmonary artery branches (Figure 5, [Supplementary-material supplementary-material-1]).

A discussion was held with the patient and his family including surgical options; however, due to the potentially high operative risks and poor baseline functional capacity, they decided to pursue medical management and focus on quality of life.

## 2. Discussion

Currently, congenital heart disease (CHD) affects more adults than children in the United States [1]. Ideally, patients with complex CHD will be referred to a specialized adult congenital heart disease clinic; however, often this is not possible, and therefore, general and interventional adult cardiologists will inexorably be involved in their care. Thus, awareness about potential challenges and limitations during diagnostic workup of this condition is needed.

The association of pulmonary atresia, VSD, and MAPCA is a complex CHD that occurs in approximately 7 to 10 of 100000 live births [2, 3]. Cyanosis, heart failure, and failure to thrive are the most common initial presentations [3]. If unoperated, it carries a significant mortality of about 40% at 1 year and 80% at 20 years [3]. Thus, encountering adults with this condition is extremely rare, with only a few reports available in the literature and the oldest described patient being 54 years old [4].

Echocardiography is insufficient to fully identify pulmonary atresia, VSD, and MAPCA; however, a dedicated congenital echocardiogram is the preferred first-line imaging in these patients. Cardiac magnetic resonance imaging and computed tomography could supplement anatomic details and even pulmonary blood flow analysis; however, due to the lack of detailed hemodynamic data in these two modalities, cardiac catheterization remains the gold standard for accurately diagnosing this condition [3].

Identification of MAPCA during cardiac catheterization is of exceptional importance, since it will determine treatment strategies. Most MAPCAs have a thoracic origin (descending aorta and subclavian arteries), but they can also arise from cervical vessels [5], the abdominal aorta and even from the coronary arteries in rare cases [6]. In our case, critical attention was paid not only to identify the pulmonary arteries but also to measure hemodynamics.

Surgical management in children could be achieved with native pulmonary artery rehabilitation followed by complete repair, MAPCA unifocalization (anastomosis of ipsilateral MAPCA via patches or conduit, incorporating the ipsilateral native pulmonary artery), or a combined strategy of the two approaches [2]. However, in adults with this condition, pulmonary hypertension may limit the feasibility of the former methods, and medical therapy, combined heart/lung transplant, or palliative care may be the only options.

## 3. Conclusions

The association of pulmonary atresia, VSD, and MAPCA is a rare complex CHD that is mainly diagnosed with cardiac catheterization. When encountered by an adult interventional cardiologist, obtaining anatomic and hemodynamic details is challenging.

## Additional Points

*Learning Objective*. This case highlights the challenges that an adult interventional cardiologist may have when encountering patients with undiagnosed congenital heart disease.

## Supplementary Materials

Supplementary 1 Video 1: distal thoracic aortogram showing collaterals originating the right upper pulmonary artery branch.

Supplementary 2 Video 2: subclavian angiogram demonstrating collaterals originating the left pulmonary artery and right lower pulmonary artery branch.
